# Seasonal variations of all-cause and cause-specific mortality by age, gender, and socioeconomic condition in urban and rural areas of Bangladesh

**DOI:** 10.1186/1475-9276-10-32

**Published:** 2011-08-04

**Authors:** Katrin Burkart, Mobarak H Khan, Alexander Krämer, Susanne Breitner, Alexandra Schneider, Wilfried R Endlicher

**Affiliations:** 1 Humboldt-Universität zu Berlin, Department of Geography, Berlin, German; 2 Universität Bielefeld, School of Public Health, Bielefeld, Germany; 3 Department of Statistics, Jahangirnagar Universtity, Savar, Bangladesh; 4 Helmholtz Zentrum München - German Research Center for Environmental Health, Institute of Epidemiology II, München, Germany

## Abstract

**Background:**

Mortality exhibits seasonal variations, which to a certain extent can be considered as mid-to long-term influences of meteorological conditions. In addition to atmospheric effects, the seasonal pattern of mortality is shaped by non-atmospheric determinants such as environmental conditions or socioeconomic status. Understanding the influence of season and other factors is essential when seeking to implement effective public health measures. The pressures of climate change make an understanding of the interdependencies between season, climate and health especially important.

**Methods:**

This study investigated daily death counts collected within the Sample Vital Registration System (VSRS) established by the Bangladesh Bureau of Statistics (BBS). The sample was stratified by location (urban vs. rural), gender and socioeconomic status. Furthermore, seasonality was analyzed for all-cause mortality, and several cause-specific mortalities. Daily deviation from average mortality was calculated and seasonal fluctuations were elaborated using non parametric spline smoothing. A seasonality index for each year of life was calculated in order to assess the age-dependency of seasonal effects.

**Results:**

We found distinctive seasonal variations of mortality with generally higher levels during the cold season. To some extent, a rudimentary secondary summer maximum could be observed. The degree and shape of seasonality changed with the cause of death as well as with location, gender, and SES and was strongly age-dependent. Urban areas were seen to be facing an increased summer mortality peak, particularly in terms of cardiovascular mortality. Generally, children and the elderly faced stronger seasonal effects than youths and young adults.

**Conclusion:**

This study clearly demonstrated the complex and dynamic nature of seasonal impacts on mortality. The modifying effect of spatial and population characteristics were highlighted. While tropical regions have been, and still are, associated with a marked excess of mortality in summer, only a weakly pronounced secondary summer peak could be observed for Bangladesh, possibly due to the reduced incidence of diarrhoea-related fatalities. These findings suggest that Bangladesh is undergoing an epidemiological transition from summer to winter excess mortality, as a consequence of changes in socioeconomic conditions and health care provision.

## Background

Seasonality of mortality and, in general, of disease is a well-known phenomenon in many regions and countries worldwide. Numerous studies have been conducted in industrialized countries of the mid-latitudes [1-7] that relate a multitude of causes of death to seasonal incidence (e.g., cardio-respiratory diseases, infectious disease) [5,8-13]. Likewise, seasonal fluctuations have been observed for tropical climates [14-18], despite the less-pronounced intra-annual climatological variation that is mainly related to seasonal differences in precipitation. Nevertheless, the number of studies focusing on tropical climates is limited, especially for Asian countries.

Although seasonal variations are to some extend driven by seasonal variations in weather, they underlie various non-atmospheric influences. These influences have fundamentally modified the shape of the seasonal pattern over recent centuries [5,19-22]. In developed countries, a shift from a summer peak in mortality towards a winter peak has been observed (ibid). In contrast, tropical countries have been, and still are, associated with excess summer mortality; this is often explained by a high prevalence of infectious and diarrhoeal disease [17,18,23-25]. The modifying effect of non-atmospheric parameters is well-demonstrated by the existence of different seasonality regimes within the same climatic region. For instance, major differences between urban and rural areas could be observed in France in the 18th century [26], and were also found between White and Afro-American groups in Philadelphia in the same century [27]. In a more recent study, education serving as a proxy for socioeconomic status has been highlighted as a determinant for seasonal fluctuations of mortality [5].

Apart from climate, cultural and behavioural aspects apparently play a major role in shaping the seasonal distribution of mortality [5,28,29]. Paradoxically, studies show that countries with a relatively warm or mild winter climate, such as Spain, Portugal, Italy, or the UK and Ireland, experience much greater excess winter mortality than countries with harsh climatic conditions during winter, such as Finland, Norway, or Russia (Siberia) (ibid). Better adjustment and social adaptation to the cold in countries with cold winter climates have been cited as explanations (ibid). There is evidence that with the same outdoor temperatures, people living in colder climates wear warmer clothes and protect themselves better against the cold [29-31].

Understanding the impact of seasonally varying factors, the effects of atmospheric conditions and the modifying effect of non-atmospheric influences can make a contribution to establishing effective public health measures. Whereas in a recent study [32] we investigated the short-term effects of thermal conditions, this study seeks to asses mid-to long-term seasonal effects and atmospheric influences. To date, few studies have investigated the atmosphere-mortality relationship in a tropical developing country; whereas the vast majority of all such research has concentrated on an assessment of seasonal effects rather than immediate meteorological effects. In focusing on seasonality we were able to set the findings of this work in the context of other studies thus allowing comparison of results. Moreover, conducting a multi-stratified analysis enabled us to reach a better understanding of the various non-atmospheric effect modifiers. In particular, we focused on the differences between urban and rural areas, gender differences and differences between regions with different socioeconomic status (SES). We also considered various causes of death and age-specific effects.

## Methods

Mortality data were provided by the Bangladesh Bureau of Statistics (BBS) for the period from 2002 to 2007. The data were collected within the Sample Vital Registration System (SVRS), which has existed in its current version since 2002. The SVRS comprises and surveys 1,000 Primary Sample Units (PSU) in rural and urban areas, and in the statistical metropolitan area (SMA). A PSU is a compact cluster with approximately 200 households with a household size of 4-5 members (4.7 on average). A number of 640 PSUs is located in rural areas, comprising 132,646 households; 280 PSUs are located in urban areas with 57,852 households and 80 PSU are placed in the SMA with 16,024 households. The SVRS provides information on housing, household, and population characteristics that are continuously updated. Data is collected using a dual recording system. Initially, vital events are collected by a locally-recruited recorder (System 1). Subsequently, data are collected in retrospective by a group of officials from the BBS on a quarterly basis (System 2). Afterwards, data are matched by pre-designed matching criteria and classified into matched, partially-matched and non-matched events. Partially-matched and non-matched events are subject to further verification through field visits. The following information about each fatal event was recorded: name of the deceased, date of birth, date of death and sex of the deceased. Moreover, a cause of death is attributed, which, however, is not medically certified. For further information see [33].

For this study all accidental deaths, maternity-related deaths, data from the statistical metropolitan area, and the data of 2002 were excluded. Furthermore, we excluded deaths of infants younger than one year, as births exhibit seasonal variations which could confound our analysis. As severe flooding submerged major parts of the country in 2004, we conducted a sensitivity analysis running our analysis with and without data from this year. Results were mostly unaffected, except for diarrhoeal mortality and other-cause mortality. Therefore, the analysis on those two causes of death was done excluding deaths occurring in 2004. In total, 21,551 deaths were analyzed. Stratified-time series plots of the daily death counts are provided in an additional file (Additional file 1).

Daily mortality was defined as daily crude death rates (number of deaths per population). In order to facilitate comparison between different subcategories we calculated the percent deviation from average mortality for each day of the year from 2003 to 2007. Average daily mortality (expected mortality) was calculated separately for each year in order to account for inter-annual changes in mortality.

In order to assess the extent of seasonality and its age-specific characteristics, we calculated a seasonality index (Φ) for each year of life, defined as the ratio of the number of deaths in the months with the highest and lowest mortality (see Formula 1).(1)

With Φ being the seasonality index, N_Max _and N_Min _the numbers of deaths in the months with the highest and lowest death counts in year *i*, and *n *the number of years from 2003 to 2007.

To display seasonal variations of mortality and age-specific seasonality of mortality we smoothed the data with penalized spline (P-spline) smoothers. Unlike many other seasonality studies we chose a non-parametric smoothing approach in order to account for multi-modal seasonal distributions. Data analysis and smoothing was carried out using R (Version 2.11.0) and the R package 'mgcv'. P-splines applied in this analysis are as proposed by Eilers and Marx [34].

The analysis of seasonality by SES was conducted on the administrative level of Zilas, which are subunits of the six divisions existing in Bangladesh. To distinguish between Zilas with high and low SES, we derived four socioeconomic factors by conducting a factor analysis (Varimax rotation) using R (Version 2.7.2). Variables considered for factor analysis were child mortality rate, child/woman ratio, literacy rate, fertility rate, source of drinking and non-drinking water, infant mortality, insolvency rate and use of solid fuels. At least three of the four derived factors being below or above the 50th percentile were criteria for Zilas to be categorized as either low or high SES. Of the 64 Zilas, 23 were categorized as having poor SES and 25 were categorized as having high SES. The remaining Zilas were treated as reference. Like in most developing countries, differences between sub-populations are strongly pronounced, but also differences between the considered divisions were marked. The relative standard deviation for the use of surface water as drinking water reached up to 400%; also for infant and child mortality or insolvency the relative standard deviations ranged between 30 and 70%.

## Results

### Causes of death

Major causes of death were respiratory diseases (~18%), cardiovascular diseases (~14%) and infectious diseases (~14%). Cancer made up approximately 6% of all deaths. Diarrhoeal disease only accounted for approximately 4%, while vector-borne diseases and malnutrition accounted for approximately 2% of all deaths on average. Approximately 18% of mortalities were classified as "old-age diseases", and approximately 22% were not specifically classified.

**Figure 1:**
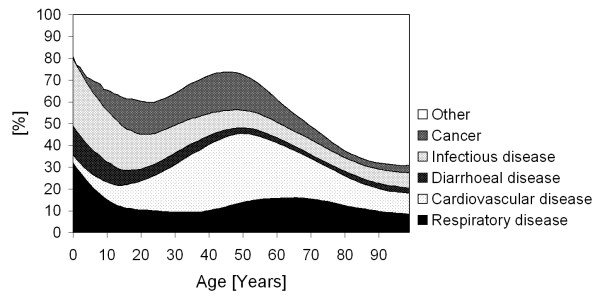
**Age-dependency of causes of death**.

The distribution of cause of death varied with age (Figure 1). The main causes of death among children were respiratory, diarrhoeal and infectious diseases. However, these causes declined for juveniles and older persons, and other causes of death, such as cancer and cardiovascular diseases, became more dominant. Besides the age-dependency of the distribution of cause of death, we found differences between urban vs. rural areas, between males vs. females, and between regions with high vs. low SES (Table 1). The Chi-square test was applied to determine whether the probability of dying from a particular disease was significantly different between two categories (rural vs. urban, male vs. female, high vs. low SES). The risk of dying from respiratory, diarrhoeal, infectious or vector-borne disease was significantly higher in rural areas, while in urban areas the risk of dying from cardiovascular disease or cancer was higher. Likewise, significant differences were found between males and females with increased probability in males of dying from respiratory or cardiovascular disease and females dying from diarrhoeal and infectious disease or malnutrition. In low SES regions a higher probability of dying from diarrhoeal disease was observed whilst in high SES regions the probability of dying from cardiovascular disease was higher.

**Table 1:** Percentage of deaths attributed to selected diseases by location, gender and socioeconomic status (SES) (2003-2007)

Cause of death	Rural	Urban	p-value*	Male	Female	p-value*	Low SES	High SES	p-value*
Respiratory disease	19.4	16.1	*<0.001*	19.4	17.0	*<0.001*	14.1	14.2	*0.95*
Cardiovascular disease	11.5	20.8	*<0.001*	16.7	11.0	*<0.001*	14.7	17.6	*<0.001*
Diarrhoeal Disease	3.9	2.7	*<0.001*	3.2	4.1	*<0.001*	3.5	2.9	*0.02*
Infectious disease	13.9	9.4	*<0.001*	12.2	13.7	*<0.001*	10.1	10.5	*0.36*
Cancer	6.2	7.5	*<0.001*	6.7	6.3	*0.16*	7.1	7.7	*0.13*
Malnutrition	2.0	1.8	*0.24*	1.7	2.4	*<0.001*	1.1	0.7	*0.10*
Vector-borne disease	1.5	0.7	*<0.001*	1.4	1.3	*0.39*	1.3	1.4	*0.05*
Other diseases	41.6	41.0	*0.01*	38.7	44.2	*<0.001*	48.1	45.0	*<0.001*
**Total**	**100**	**100**		**100**	**100**		**100**	**100**	

*: p-values were determined by Chi-square tests with a significance level of 0.05.

### Seasonal variations of all-cause mortality and cause-specific mortality

**Figure 2:**
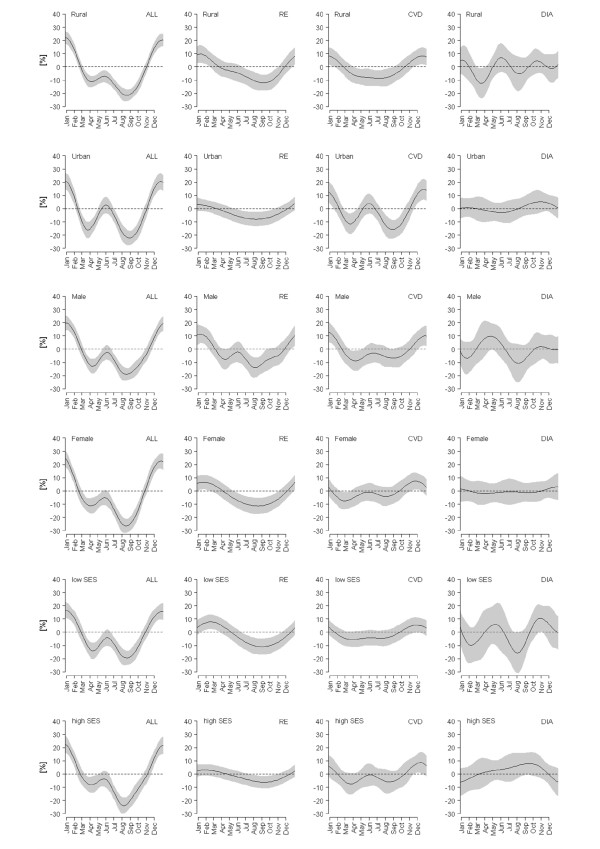
**Seasonal mortality variations of all-cause mortality (ALL), respiratory mortality (RE), cardiovascular mortality (CVD), and diarrhoeal mortality (DIA) distinguished between different subcategories (rural vs. urban, male vs. female, low vs. high SES)**. (The 95%-confidence intervals are displayed by the shaded areas).

All-cause and cause-specific mortality showed a pronounced seasonality, except for cancer. For our further analysis of cause-specific seasonality we focused on respiratory, cardiovascular and diarrhoeal mortality. Analyses for other causes of death are included in an additional file (Additional file 2), but will not be discussed due to the complexity of disease pathogenesis and multitude of causative agents, particularly in the case of infectious and other disease mortality. All-cause mortality exhibited a marked seasonality with a bimodal distribution. The primary maximum occurred during the cold and dry season (October to February), and the secondary maximum was from May to July, at the end of the summer season and the beginning of the monsoon season (Figure 2). With regard to different disease groups, marked seasonal variations could also be observed, with the shape of the seasonal distribution being determined by location, gender, or SES. Generally, respiratory and cardiovascular mortality were highest during the cold season, with some strata additionally showing increased levels during summer. Seasonal fluctuations in diarrhoeal mortality varied heavily by investigated category, with no seasonal pattern or multiple seasonal peaks and troughs occurring during the year.

### Seasonal variation by location, gender, and SES

A greater excess of all-cause mortality in summer was evident in urban areas than in rural areas, while winter excess mortality was equally high in both areas (Figure 2). For females and regions with high SES slightly stronger seasonal fluctuations were observed, originating mainly from a strong negative deviation of mortality during the monsoon season. Deaths from respiratory diseases generally peaked during the cold season, with a small secondary maximum during summer for males (Figure 2). In urban and high SES areas, respiratory seasonality was only minor. Cardiovascular mortality was generally higher during the cold winter season. Nevertheless, urban areas exhibited a pronounced secondary summer maximum of cardiovascular mortality and in areas with high SES, a small summer maximum was observed as well (Figure 2).

Diarrhoeal mortality showed the most complex seasonal variations. In general, two maxima occurred: one between the summer and the beginning of the monsoon season, and the other at the end of the monsoon season. However, size and exact temporal occurrence varied with location, gender and SES (Figure 2). In rural areas a primary peak from April to June occurred and a secondary peak at the end of the rainy season. Additionally, rural diarrhoeal mortality was increased during winter, in December and January. In urban areas some sort of a post-monsoon maximum, with slightly increased levels from September to December was observed. However, variations in urban areas were only minor (Figure 2). For males a marked primary peak from March to June and a less marked secondary maximum at the end of the monsoon season were detected. In the case of females, no seasonal pattern of diarrhoeal mortality emerged. In regions with low SES, two equally pronounced maxima occurred: one in summer and one at the end of the monsoon season. In regions with high SES, diarrhoeal mortality was slightly increased at the end of the monsoon season and diminished during winter.

### Age-dependency of seasonality

**Figure 3:**
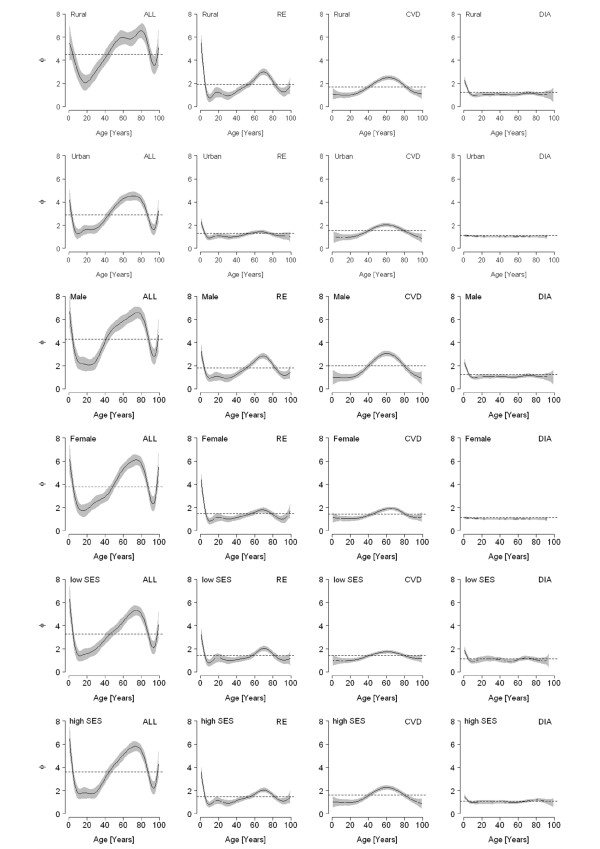
**Age-dependency of seasonality of all-cause mortality (ALL), respiratory mortality (RE), cardiovascular mortality (CVD), and diarrhoeal mortality (DIA) distinguished between different subcategories (rural vs. urban, male vs. female, low vs. high SES)**. (Mean of seasonality index is indicated by the dashed lines. The 95%-confidence intervals are displayed by the shaded area).

For all considered diseases, the magnitude of seasonality represented by the seasonality index (Φ) was strongly age-dependent (Figure 3). Generally, children faced a high magnitude of seasonality while for youths and young adults seasonality was less important. With progressing age, the magnitude of seasonality rose again and reached maximum levels at the age of 60 to 80 years. Above this age, seasonality declined again. Respiratory seasonality was strongly pronounced for children and additionally pronounced for older age groups between 40 and 80. Particularly, children and the elderly in rural areas, as well as elderly males, faced a strong seasonal risk. Cardiovascular seasonality played no role for children, but was pronounced for persons aged between 40 and 80 years, peaking at the age of 60. Predominantly males and regions with high SES were subject to strong seasonal effects on cardiovascular mortality in the elderly. In the case of diarrhoeal mortality, seasonality mainly played a role for children in their first years of life and then rapidly subsided. Diarrhoeal child mortality was particularly subject to seasonality in rural areas and in males.

## Discussion

In this study we identified distinct seasonal variations of mortality, with the shape of the seasonal pattern depending on the cause of death, age, gender, and location, as well as SES. Unlike findings from other tropical regions [17,18,23-25,35-39], we did not find a primary maximum in mortality during the hot and humid/rainy season. In our data the summer excess mortality may have been represented by the secondary maximum from April to July. However, the main peak in mortality occurred during the cold season. This observation suggests that Bangladesh is undergoing an epidemiological transition as the incidence of diarrhoeal deaths reduces considerably and cardio-respiratory diseases become more dominant. A transition in the seasonal pattern - from a summer to a winter peak - has also been described for Europe and Japan, and SES changes and improved medical care served as explanations [5,21]. Indeed, a study analysing death counts from 1972-1974 from a rural area in Bangladesh found a winter maximum for dysentery and chronic diarrhoeal mortality. The author ascribed this pattern to the high quality of diarrhoeal health care in that particular (intervention) area and acknowledged that such a mortality pattern is possibly not found in other areas [14]. In a follow-up study, in which data from 1982 to 1990 was analyzed, a winter maximum was found for all-cause mortality; however, diarrhoeal deaths peaked during the hot and humid season [15]. To date, the only lower-latitude regions exhibiting a mortality peak during the cold season are Mexico City (Mexico) and São Paulo (Brazil), both located within higher altitudes and exhibiting temperate climatic conditions (type C-climates according the Köppen-Geiger classification) as well as a generally higher SES [16]. In addition, a higher mortality during the cold months was found for under-five mortality in Nairobi (Kenya), also classified as a temperate type C-climate [40,41]. To our knowledge, the only country with a tropical type A-climate for which a winter excess mortality has been established is Bangladesh.

Nowadays, respiratory and cardiovascular diseases are the leading causes of death in Bangladesh. We found that both diseases as well as all-cause mortality peaked during winter, despite the perception of the relatively cold season as being thermally comfortable. These findings suggest that winter excess mortality is not a consequence of seasonal low absolute temperatures but, rather, is a consequence of a seasonal fall in average temperature. The adverse effect of relatively low temperatures was also demonstrated in a recent study using the same data set [32]. Regression models adjusted for the confounding effects of trend, season and day of the month revealed an increase in mortality with temperatures falling below a threshold situated in the 90^th ^to 95^th ^percentile of the temperature distribution. While adequately adapted to heat, the Bangladeshi population seems less prepared for the winter period. The importance of relative cold is also highlighted by research conducted by Douglas et al. [1] and the Eurowinter Group [42].

The pronounced summer peak of all-cause and cardiovascular mortality in urban areas is possibly related to the urban excess (equivalent) temperature, the so-called urban heat island. Excess temperatures amounting to several Kelvin in monthly averages were found in urban areas of (sub)tropical regions [43,44]. Nevertheless, a higher susceptibility of the urban population towards heat might also be an explanation. A higher susceptibility towards heat might also be the cause of the secondary summer maximum of cardiovascular mortality in regions with high SES.

In this study the complexity and dynamic nature of diarrhoeal disease pathogenesis is once more illustrated. Several agents that induce diarrhoeal disease favour different meteorological and hydrological conditions for their replication and survival. High temperatures generally promote the growth of bacteria, whereas, for instance, *shigella *epidemics show a high incidence rate during the cold season [45]. Furthermore, there is evidence that moderate temperatures favour certain agents, such as the rotavirus [46,47]. Additionally, hydrological aspects, such as stagnant water, a lack of dilution, contamination, or the break down of water systems associated with no, high, or ongoing rainfall, determine the spread of diarrhoeal pathogens [46,48]. Cholera incidence showed a bimodal distribution in Bengal that seemed to be driven by hydroclimatological factors [49,50]. Low precipitation and low river discharge during spring was associated with the first outbreaks of cholera in Bangladesh whereas high precipitation and peak streamflow of rivers during the monsoon season was associated with the second peak during the monsoon season (ibid).

Given this background information, the interpretation of our findings is complex. Nevertheless, we suggest that the summer peak in diarrhoeal mortality is predominantly caused by bacterial contamination during the hot season favouring replication of agents, whilst the post-monsoonal peak probably results from overstrained sewage systems, flooding and stagnant water. The pronounced seasonal pattern in rural areas and areas with low SES is possibly due to reduced coping abilities in these regions. The absence of a summer peak in urban areas and the reduced post-monsoonal peak might be due to improved access to fresh aliments, cooling systems as well as more developed drainage and sewage systems. Regarding female diarrhoeal mortality, the nonexistence of seasonal fluctuations is surprising as females are facing an increased risk of dying from diarrhoeal mortality. Women are usually bound to their homes consuming home made food, whilst men are commonly pursuing an income generating activity outside that forces them to eat food either self-carried or purchased at some sort of restaurant. Particularly, during the summer season the risk of bacterial contamination is thus increased. In areas with high SES, higher sanitary standards, improved water and drainage systems and higher levels of education might have resulted in reduced diarrhoeal seasonality.

Our analysis further showed that seasonality is strongly age-dependent. Children in their first years of life exhibited a high magnitude of seasonality, demonstrating their susceptibility towards environmental conditions. This susceptibility to season and environment was even more pronounced in rural areas, probably resulting from poor primary health services as well as poor living standards. Although young and middle-aged adults are likely to be most excessively exposed to environmental influences they were the only group to show weak or no seasonality. The increasing seasonality index with increasing age reflects the growing susceptibility of the human organism towards environmental conditions. The observed decrease in seasonality between the ages of 80 and 90 might be due to the so called selection effect, described in detail by Rau [5]. Briefly, the phenomenon is the consequence of a heterogeneous population in which frail individuals face higher mortality, leaving a robust subset. This robust subset is less susceptible to seasonal effects, lowering seasonality up to a certain age beyond which individuals once again become more susceptible.

### Strengths and Limitations

Compared to studies conducted in Western countries, our data comprise only a sample and not a complete inventory. Nevertheless, in the context of a developing country such data availability is rather exceptional as the data set includes continuous data and covers Bangladesh on a nationwide level. Incompleteness, underreporting and the absence of validation and correction of known bias are often associated with civil registration systems in developing countries [51,52]. Due to the dual recording system the authors appraise the reliability of the data as rather high. Although in other cases difficulties are imposed by the fact that many deaths occurring at home in resource-poor countries are not registered [53], here this is bypassed by the registration system, which surveys households. However, some limitations should be mentioned. One of the limitations is related to the lack of information about the socioeconomic composition of the sample. Bangladeshi society consists of a wide range of different groups with different socioeconomic and educational backgrounds. Furthermore, the data collection and registration were not medically certified and cause of death classification is rather rough and does not follow International Classification of Disease standards. Our analysis revealed that the magnitude of seasonality is strongly age-dependent; however, this finding does not allow conclusions about the temporal occurrence of maximum or minimum mortality. It may well be that the seasonal pattern reveals to be modified for different age groups with temporally displayed peaks and troughs compared to the mean seasonal pattern. The number of observations, however, did not allow a further age stratification of the seasonality plots, particularly not in the case of cause-specific mortality. Finally, we need to acknowledge that due to the nature of this ecological study which is based on aggregated information, inferences need to be made carefully as the risk of ecological fallacy is implied.

## Conclusions

This study demonstrated the importance of considering seasonal impacts on mortality and revealed several target groups and diseases for which the consideration of seasonality seems particularly crucial. We showed that the effect of season or seasonally changing environmental conditions depends on preconditions in a subpopulation or region. In particular, rural areas showed a high magnitude of infant and child seasonality, while urban areas were strongly associated with summer excess mortality, especially for cardiovascular mortality. Children and elderly people were affected most by seasonal effects. The consideration of such seasonal effects could help to place public health interventions most effectively. Generally, protection measures against cold or heat among infants or those under hospital care can help to avoid or overcome critical states of health. We emphasise that knowledge on the interaction between seasons, atmosphere, and health is especially necessary in times of climate change so that its possible impacts can be mitigated.

## Competing interests

The authors declare that they have no competing interests.

## Authors' contributions

KB developed the analytical approach, performed the statistical analysis, and drafted the manuscript. WRE, MHK and AS assisted with the analytical approach and contributed to the drafting of the manuscript. SB and AS contributed to the analysis and drafting of the manuscript. All authors have read and approved the final manuscript.

## Supplementary Material

Additional file 1 **Stratified-time series plots of daily death counts**. Daily death counts from 2003 to 2007 stratified by location, gender, and SES and smoothed with penalized splines. Click here for file

Additional file 2 **Seasonality analysis for infectious disease, cancer, and other-cause mortality**. Seasonal mortality variations and age-dependency of infectious disease seasonality (INF), cancer seasonality (CAN), and other-disease seasonality (OTH) distinguished between different subcategories (rural vs. urban, male vs. female, low vs. high SES). Click here for file

## References

[B1] Douglas A Rawles J Al-Sayer H Allan T Seasonality of disease in Kuwait Lancet 1991 337 1393 1397 10.1016/0140-6736(91)93069-L 1674772

[B2] Gemmell I McLoone P Boddy F Dickinson G Watt G Seasonal variation in mortality in Scotland Int J Epidemiol 2000 29 274 279 10.1093/ije/29.2.274 10817125

[B3] Hernández M García-Moro C Seasonal distribution of mortality in Barcelona (1983-1985) Antropologia portuguesa 1986 4-5 211 223

[B4] Mackenbach JP Kunst AE Looman CWN Seasonal variation in mortality in the Netherlands J Epidemiol Community Health 1992 46 261 265 10.1136/jech.46.3.261 1645083 PMC1059564

[B5] Rau R Seasonality in Human Mortality. A Demographic Approach 2006 Berlin: Springer

[B6] Rosenwaike I Seasonal variation of deaths in the United States, 1951-1960 J Am Stat Assoc 1966 61 706 719 10.2307/2282781

[B7] Feinstein CA Seasonality of deaths in the U.S. by age and cause Demogr Res 2002 6 469 486

[B8] Cadet B Robine J Leibovici D Dynamique de la mortalité asthmatique en France: fluctuations saisonnières et crise de mortalité en 1985-87 Revue d'Epidémiologie et de Santé Publique 1994 42 103 118 8184154

[B9] Chung-Jen Y Chia-Lun C Fung-Chang S Wen-Jone C Chiau-Suong L Lee Y Seasonal effects on cardiovascular mortality in older patients Age Ageing 2000 29 186 187 10791461 10.1093/ageing/29.2.186b

[B10] Momiyama M Changes in seasonality of human deaths from infectious diseases 1987 The Hague: SPB Academic Publishing

[B11] Villa S Guisecafré H Martinez H Muñoz O Seasonal diarrhoeal mortality among Mexican children Bull World Health Organ 1999 77 375 380 10361753 PMC2557672

[B12] Bouma M Pascual M Seasonal and interannual cycles of endemic cholera in Bengal 1891-1940 in relation to climate and geography Hydrobiologica 2001 460 147 156 10.1023/A:1013165215074

[B13] Akanda A Jutla A Islam S Dual peak cholera transmission in Bengal Delta: A hydroclimatological explanation Geophysical Research Letters 2009 36 L19401

[B14] Becker S Seasonality of Deaths in Matlab, Bangladesh Int J Epidemiol 1981 10 271 280 10.1093/ije/10.3.271 7287288

[B15] Becker S Weng S Seasonal Patterns of Deaths in Matlab, Bangladesh Int J Epidemiol 1998 27 814 823 10.1093/ije/27.5.814 9839738

[B16] McMichael AJ Wilkinson P Kovats RS Pattenden S Hajat S Armstrong B Vajanapoom N Niciu EM Mahomed H Kingkeow C International study of temperature, heat and urban mortality: the "ISOTHURM" project International Journal of Epidemiology 2008 37 1121 1131 10.1093/ije/dyn086 18522981

[B17] Motohashi Y Takano T Nakamura K Nakata K Tanaka M Seasonality of mortality in Sri Lanka: Biometeorological considerations International Journal of Biometeorology 1996 39 121 126 10.1007/BF01211223 8984066

[B18] Ruzicka L Kanitkar T Infant mortality in Greater Bombay Demography India 1973 II 41 55

[B19] Keatinge W Coleshaw S Holmes J Changes in seasonal mortalities with improvement in home heating in England and Wales from 1964 to 1984 Int J Biometeorol 1989 33 71 76 10.1007/BF01686280 2759722

[B20] Kunst A Looman C Mackenbach J The Decline in Winter Excess Mortality in the Netherlands Int J Epidemiol 1990 20 971 977 10.1093/ije/20.4.971 1800438

[B21] Sakamoto-Momiyama M Changes in the Seasonality of Human Mortality: A Medico-Geographical Study Soc Sci Med 1978 12 29 42 10.1016/0160-8002(78)90005-9 644348

[B22] Seretakis D Lagiou P Lipworth L Signorello L Rothman K Trichopoulos D Changing Seasonality of Mortality from Coronary Heart Disease JAMA 1997 278 1012 1014 10.1001/jama.278.12.1012 9307350

[B23] Underwood J Seasonality of vital events in a Pacific Island population Social Biology 1991 38 113 126 1749959 10.1080/19485565.1991.9988775

[B24] Jaffar S Leach A Greenwood A Jepson A Muller O Ota M Bojang K Obaro S Greenwood B Changes in the pattern of infant and childhood mortality in Upper River Division, The Gambia, from 1989 to 1993 Tropical Medicine & International Health 1997 2 28 37 10.1046/j.1365-3156.1997.d01-131.x 9018300

[B25] Madrigal L Mortality seasonality in Escazú, Costa Rica, 1851-1921 Human Biology 1994 66 433 452 8026814

[B26] Bideau A Dupâquier J Gutierrez H La mort quantifiée 1988 Paris: Presses Universitaires de France

[B27] Klepp S Seasoning and Society: Racial Differences in Mortality in Eighteenth-Century Philadelphia William Mary Q 1994 51 473 506 10.2307/2947439

[B28] The Eurowinter Group Winter mortality in relation to climate International Journal of Circumpolar Health 2000 59 154 159 11209660

[B29] The Eurowinter Group Cold exposure and winter mortality from ischemic heart disease, respiratory disease, and all causes in warm and cold regions of Europe The Lancet 1997 349 1341 1346 9149695

[B30] Donaldson G Ermakov S Komarov Y McDonald C Keatinge W Cold related mortalities and protection against cold in Yakutsk eastern Siberia: observation and interview study BMJ 1998 317 978 982 9765165 10.1136/bmj.317.7164.978 PMC28681

[B31] Donaldson G Tchernjavskii V Ermakov S Bucher K Keatinge W Winter mortality and cold stress in Yekaterinburg, Russia: interview survey British Medical Journal 1998 316 514 518 9501713 10.1136/bmj.316.7130.514 PMC2665668

[B32] Burkart K Breitner S Schneider A Khan M Krämer A Endlicher W The effect of atmospheric thermal conditions and urban thermal pollution on all-cause and cardiovascular mortality in Bangladesh Environmental Pollution 2011 159 2035 2043 10.1016/j.envpol.2011.02.005 21377776

[B33] Bangladesh Bureau of Statistics (BBS) Report on the Sample Vital Registration System 2007 Book Report on the Sample Vital Registration System 2007 2008 City

[B34] Eilers P Marx B Flexible Smoothing with B-splines and Penalties Stat Sci 1996 11 89 121 10.1214/ss/1038425655

[B35] Victoria C Vaughan J Barros F Seasonality of infant deaths due to diarrheal and respiratory diseases in Southern Brazil, 1974-1978 Bulletin of the Pan American Organisation 1985 19 29 39 4027452

[B36] McGregor IA Billewicz WZ Thomson AM Growth and Mortality in Children in an African Village British Medical Journal 1961 2 1661 1666 10.1136/bmj.2.5268.1661 20789304 PMC1970800

[B37] Rayco-Solon P Moore SE Fulford AJ Prentice AM Fifty-year mortality trends in three rural African villages Tropical Medicine & International Health 2004 9 1151 1160 10.1111/j.1365-3156.2004.01325.x 15548310

[B38] Delaunay V Etard J-Fo Préziosi MP Marra A Simondon Fo Decline of infant and child mortality rates in rural Senegal over a 37-year period (1963-1999) International Journal of Epidemiology 2001 30 1286 1293 10.1093/ije/30.6.1286 11821330

[B39] Etard JF Le Hesran J-Y Diallo A Diallo JP Ndiaye JL Delaunay V Childhood mortality and probable causes of death using verbal autopsy in Niakhar, Senegal, 1989-2000 International Journal of Epidemiology 2004 33 1286 1292 10.1093/ije/dyh259 15569662

[B40] Ye Y Zulu E Mutisya M Orindi B Emina J Kyobutungi C Seasonal pattern of pneumonia mortality among under-five children in Nairobi's informal settlements The American Journal of Tropical Medicine and Hygiene 2009 81 770 775 10.4269/ajtmh.2009.09-0070 19861609

[B41] Mutisya M Orindi B Emina J Zulu E Ye Y Is mortality among under-five children in Nairobi slums seasonal? Tropical Medicine & International Health 2010 15 132 139 19883400 10.1111/j.1365-3156.2009.02419.x

[B42] The Eurowinter Group Cold exposure and winter mortality from ischemic heart disease Lancet 1997 349 1341 1346 9149695

[B43] Roth M Review of urban climate research in (sub)tropical regions International Journal of Climatology 2007 27 1859 1873 10.1002/joc.1591

[B44] Burkart K Endlicher W Krämer A, Khan M, Kraas F Human Bioclimate and Thermal Stress in the Megacity of Dhaka, Bangladesh - A Climatological Approach to Health Relevance Assessment Health in Megacities and Urban Areas 2011 Heidelberg: Springer

[B45] Hossain M Albert M Hasan K Epidemiology of Shigellosis in Teknaf, a Coastal Area of Bangladesh: A 10-year Survey Epidemiol Infect 1990 105 41 49 10.1017/S0950268800047622 2200700 PMC2271791

[B46] Hashizume M Armstrong B Hajat S Wagatsuma Y Faruque ASG Hayashi T Sack DA Association between climate variability and hospital visits for non-cholera diarrhoea in Bangladesh: effects and vulnerable groups Int J Epidemiol 2007 36 1030 1037 10.1093/ije/dym148 17664224

[B47] Levy K Hubbard A Eisenberg J Seasonality of rotavirus disease in the tropics: a systematic review and meta-analysis Int J Epidemiol 2008 38 1487 1496 19056806 10.1093/ije/dyn260 PMC2800782

[B48] Zhang Y Bi P Hiller J Sun Y Ryan P Climate Variations and Bacillary Dysentery in Northern and Southern Cities of China J Infect 2007 55 194 200 10.1016/j.jinf.2006.12.002 17258812

[B49] Akanda AS Jutla AS Islam S Dual peak cholera transmission in Bengal Delta: A hydroclimatological explanation Geophys Res Lett 2009 36 L19401

[B50] Hashizume M Wagatsuma Y Faruque ASG Hayashi T Armstrong B Climatic Components of Seasonal Variation in Cholera Incidence Epidemiology 2009 20 S153 110.1097/1001.ede.0000362523.0000313023.0000362565 10.1097/EDE.0b013e3181e5b053 20562706

[B51] Huy T Long N Hoa D Byass P Ericksson B Validity and completeness of death reporting and registration in a rural district of Vietnam Scand J Public Health 2003 62 12 18 10.1080/14034950310015059 14640146

[B52] Mahapatra P Shibuya K Lopez A Coullare F Notzon F Chalapati Rao C Simon Szreter S Civil registration systems and vital statistics: successes and missed opportunities Lancet 2007 370 1653 1663 10.1016/S0140-6736(07)61308-7 18029006

[B53] Setel P Sankoh O Rao C Velkoff V Mathers C Gonghuan Y Hemed Y Jha P Lopez A Sample registration of vital events with verbal autopsy: a renewed commitment to measuring and monitoring vital statistics Bull World Health Organ 2005 83 611 617 16184280 PMC2626308

