# Supplementary material for: Seasonal variations of all-cause and cause-specific mortality by age, gender, and socioeconomic condition in urban and rural areas of Bangladesh
Source: Int J Equity Health. 2011 Aug 4;10:32. doi: 10.1186/1475-9276-10-32 (PMC3167758; doi:10.1186/1475-9276-10-32)
Supplement: Additional file 1 — Stratified-time series plots of daily death counts. Daily death counts from 2003 to 2007 stratified by location, gender, and SES and smoothed with penalized splines. [file 1475-9276-10-32-S1.DOC]

| 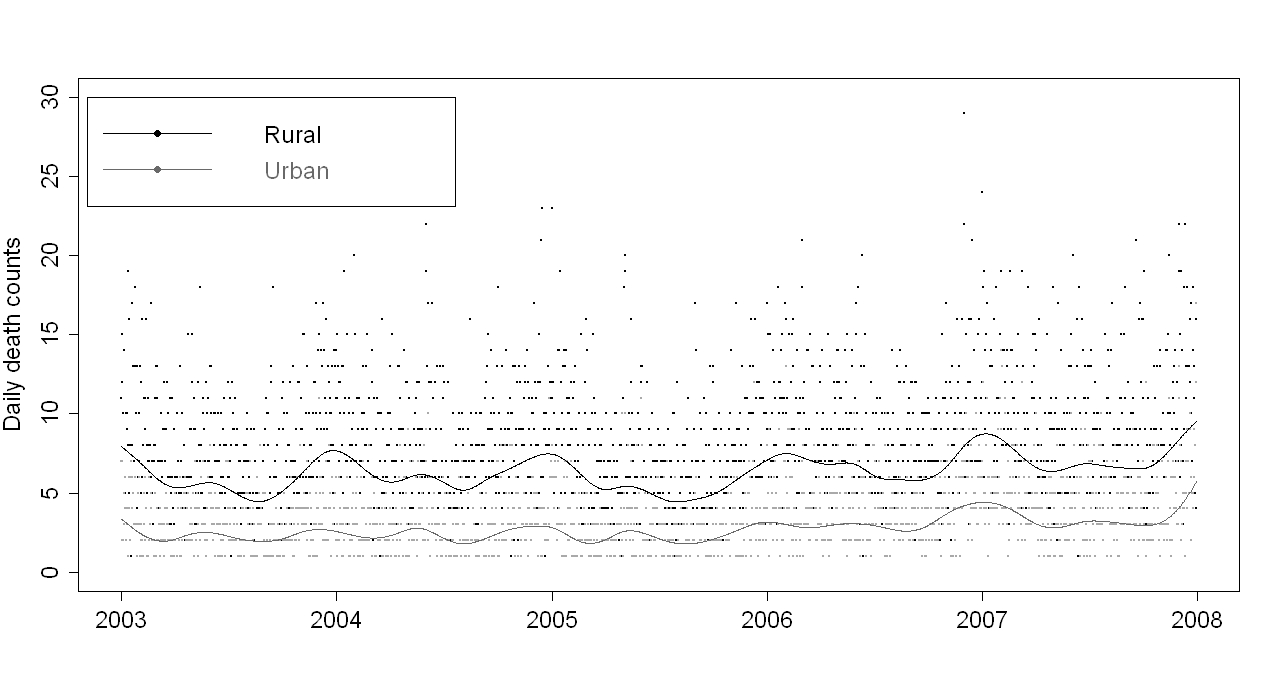 |
| --- |
| **Figure S1a. Daily death counts in rural (black) and urban (gray) areas from 2003 to 2007 smoothed with penalized splines.** |


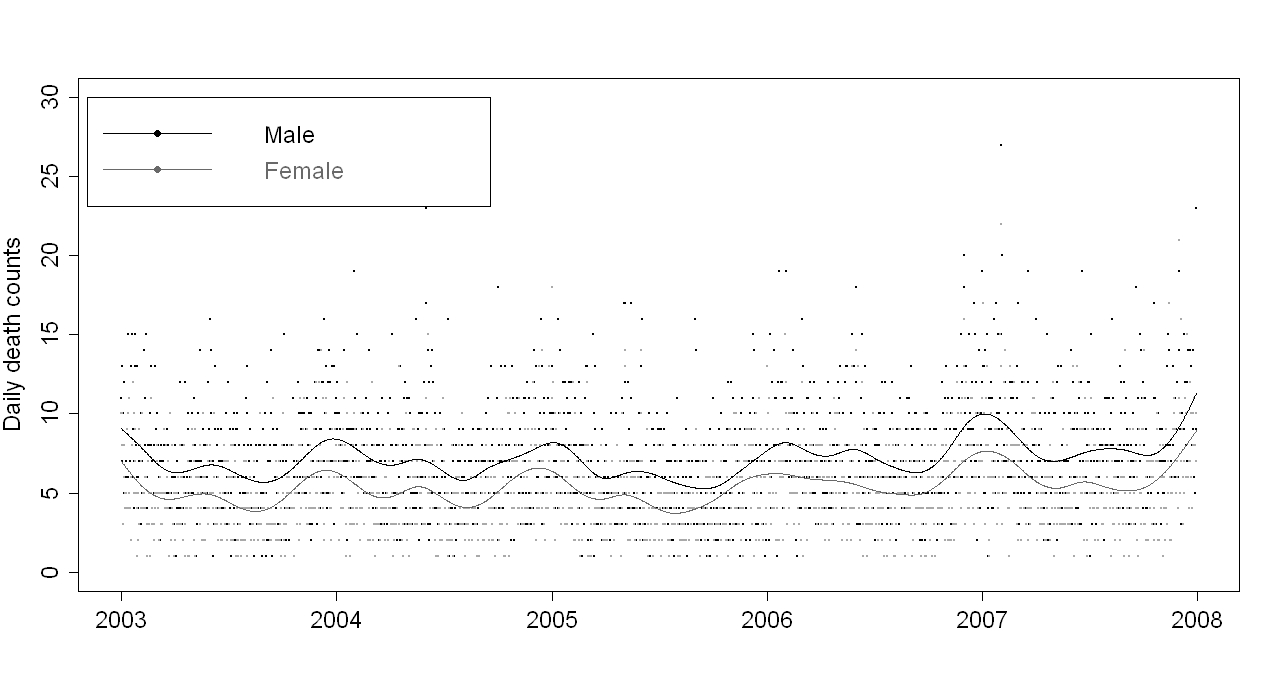


|  |
| --- |
| **Figure S1b. Daily death counts for males (black) and females (gray) from 2003 to 2007 smoothed with penalized splines.** |


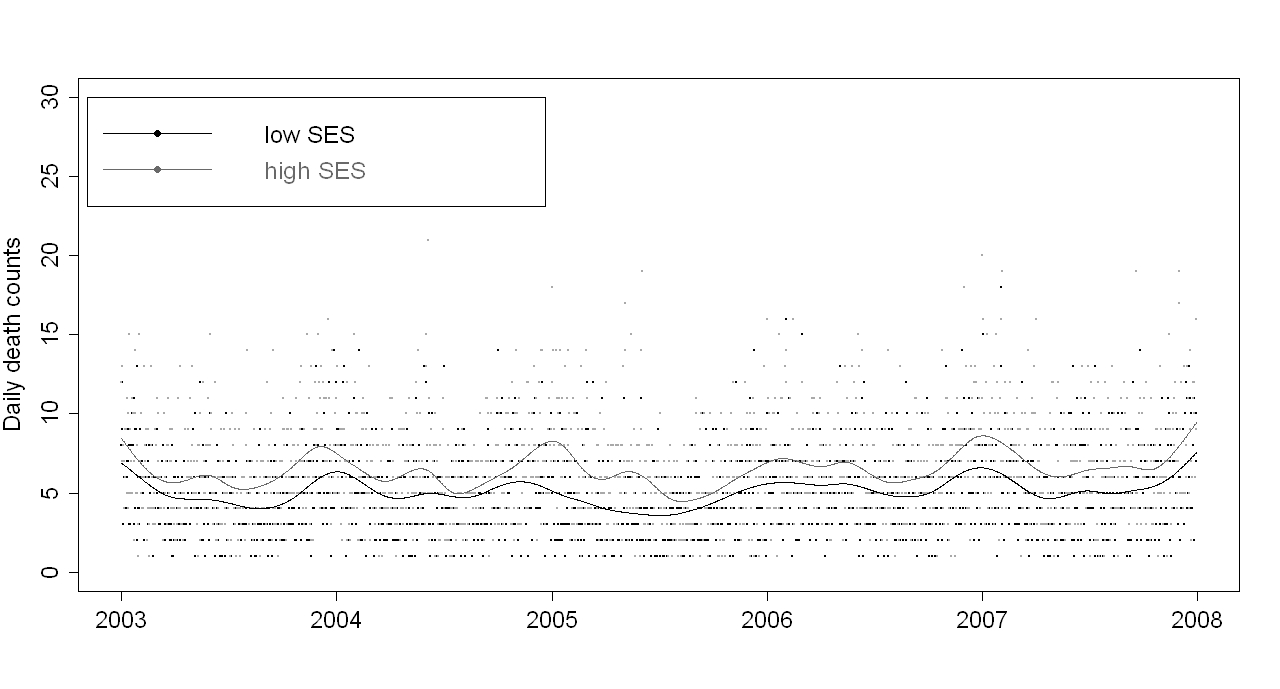


|  |
| --- |
| **Figure S1c. Daily death counts for low SES (black) and high SES (gray) from 2003 to 2007 smoothed with penalized splines.** |
