# Supplementary material for: Seasonal variations of all-cause and cause-specific mortality by age, gender, and socioeconomic condition in urban and rural areas of Bangladesh
Source: Int J Equity Health. 2011 Aug 4;10:32. doi: 10.1186/1475-9276-10-32 (PMC3167758; doi:10.1186/1475-9276-10-32)
Supplement: Additional file 2 — Seasonality analysis for infectious disease, cancer, and other-cause mortality. Seasonal mortality variations and age-dependency of infectious disease seasonality (INF), cancer seasonality (CAN), and other-disease seasonality (OTH) distinguished between different subcategories (rural vs. urban, male vs. female, low vs. high SES). [file 1475-9276-10-32-S2.DOC]

| 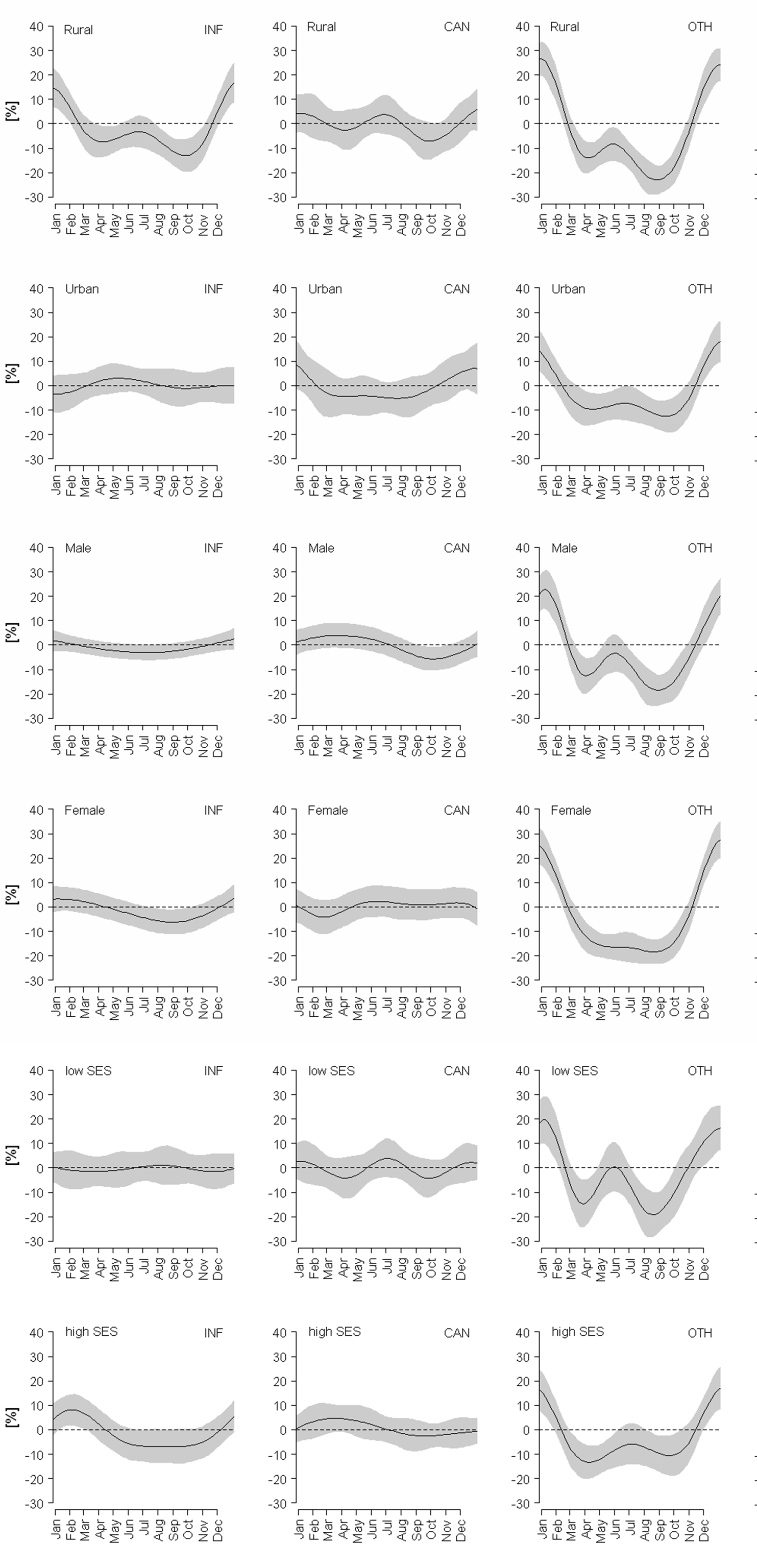 |
| --- |
| **Figure S2. Seasonal mortality variations of infectious disease mortality (INF), cancer mortality (CAN), and other disease mortality (OTH) distinguished between different subcategories (rural vs. urban, male vs. female, low vs. high SES). (The 95%-confidence intervals are displayed by the shaded areas).** |
| 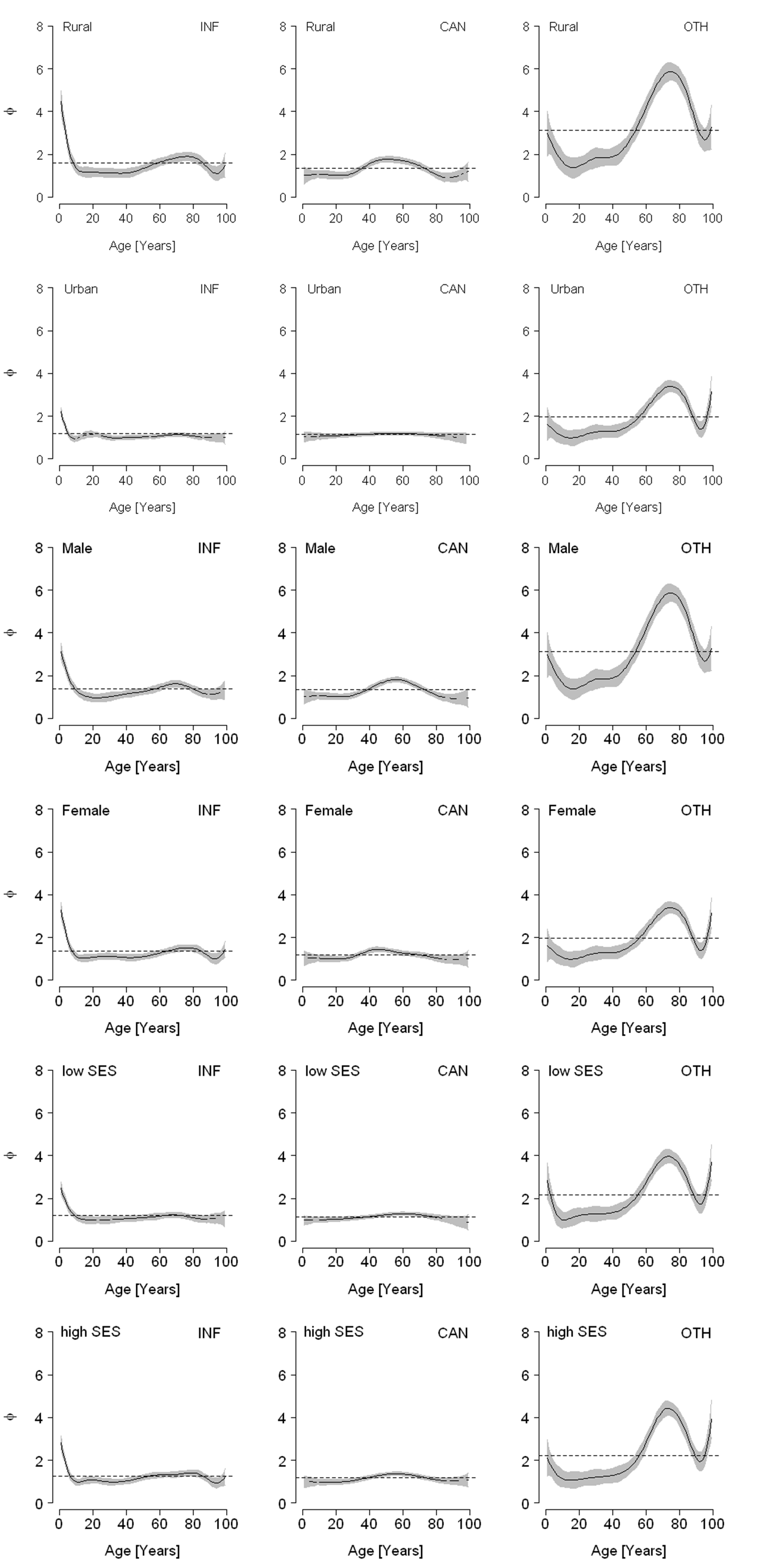 |
| **Figure S3. Age-dependency of seasonality of infectious disease mortality (INF), cancer mortality (CAN), and other disease mortality (OTH) distinguished between different subcategories (rural vs. urban, male vs. female, low vs. high SES). (Mean of seasonality index is indicated by the dashed lines. The 95%-confidence intervals are displayed by the shaded areas).** |
